# Low-Resolution Models for the Interaction Dynamics of Coated Gold Nanoparticles with β2-microglobulin

**DOI:** 10.3390/ijms20163866

**Published:** 2019-08-08

**Authors:** Giorgia Brancolini, Hender Lopez, Stefano Corni, Valentina Tozzini

**Affiliations:** 1 Istituto Nanoscienze, CNR-NANO S3, via G. Campi 213/A, 41125 Modena, Italy; 2 School of Physics and Optometric & Clinical Sciences, Technological University Dublin, Kevin Street, Dublin D08 NF82, Ireland; 3 Dipartimento di Scienze Chimiche, Università di Padova, 35131 Padova, Italy; 4 Istituto Nanoscienze—National Research Council (CNR) and National Enterprise for nanoScience and nanoTechnology (NEST) Scuola Normale Superiore, Piazza San Silvestro 12, 56127 Pisa, Italy

**Keywords:** molecular dynamics, multiscale modeling, amyloid proteins, functionalized metal nanoparticles, coarse-grained models

## Abstract

A large number of low-resolution models have been proposed in the last decades to reduce the computational cost of molecular dynamics simulations for bio-nano systems, such as those involving the interactions of proteins with functionalized nanoparticles (NPs). For the proteins, “minimalist” models at the one-bead-per residue (Cα-based) level and with implicit solvent are well established. For the gold NPs, widely explored for biotechnological applications, mesoscale (MS) models treating the NP core with a single spheroidal object are commonly proposed. In this representation, the surface details (coating, roughness, etc.) are lost. These, however, and the specificity of the functionalization, have been shown to have fundamental roles for the interaction with proteins. We presented a mixed-resolution coarse-grained (CG) model for gold NPs in which the surface chemistry is reintroduced as superficial smaller beads. We compared molecular dynamics simulations of the amyloid β2-microglobulin represented at the minimalist level interacting with NPs represented with this model or at the MS level. Our finding highlights the importance of describing the surface of the NP at a finer level as the chemical-physical properties of the surface of the NP are crucial to correctly understand the protein-nanoparticle association.

## 1. Introduction

In the last few years, several different types of nanoparticles (NPs) have been considered as therapeutic agents due to their capability of interfering with the proteins’ activity [1,2]. Particularly interesting are the gold NPs [3,4], allowing covalent versatile functionalization via thiol chemistry [5]. Thiol-protected gold NPs functionalized with phenyl groups, have been considered capable of interfering with protein aggregation [6,7], the biomolecular process pivotal in amyloidosis [8]. Their action depends on the size and chemical functionalization, which change the properties of the NP (e.g., hydrophobicity, global charge, size, and in part shape) both globally and locally. The rational optimization of the size and decoration of the NPs for the therapeutic use would greatly benefit of computer modeling of the NP-protein interaction [9,10], which, however, requires large-scale simulations of crowded particles ensembles at different concentrations. While the single or few NPs-proteins interactions are routinely feasible, the extensive exploration of different NPs size, decoration, and (relative) NPs/protein concentrations calls into play the use of low-resolution models for both interacting components.

For the protein component, the natural structural organization has suggested several kinds of residue-level coarse-grained (CG) models [11,12]. The maximum computational advantage is obtained with Cα single bead per amino-acid models with implicit solvent, also called “minimalist” [13,14], previously used and tested with a large number of different proteins [15,16] even in interaction with coarse representations of the cytoplasm component [17]. The available CG models for NPs, conversely, are rather sparse and diverse. The presence of the gold core suggests treating it at the mesoscale (MS) as a single spheroidal object [18], while the specificity of the chemical decoration [19,20] have fundamental roles for the interaction with proteins and must be treated at a higher resolution [10,21,22,23], to account for the hydrophobicity and/or electric charge borne by functionalizing groups. 

In this work, we reported molecular dynamics simulations of the interaction between the amyloidogenic β2-microglobulin (β2μ) and the phenylated gold-core NP Au_25_L_18_ (L=S(CH_2_)_2_Ph), performed with coarse-grained models. The protein was represented with the minimalist one-bead per residue model, while two different representations of the NP were considered: (i) a mesoscale one (MS) [24], with the NP represented with a single negatively charged sphere and (ii) a more refined model with a single sphere for the gold core (positively charged) decorated with residue-level beads representing the functionalizing groups (negatively charged), which we simply named coarse-grained (CG). We compared simulations from the two different resolution representations of the NP, and validated them based on atomistic simulations, to show how far coarse-graining can be pushed to the aim of extending the size and time scale of simulations. Simplified representations were also adopted for the time evolution of the system: besides standard molecular dynamics (MD) simulations, we used (and compared) Brownian dynamics (BD) associated to rigid body representations and implicit solvent. The next section reports the detail of the used methods. A section of results illustration follows, while comparison among different methods is reported in the subsequent discussion section. Conclusions and perspective are finally illustrated. 

## 2. Results and Discussion

### 2.1. Binding Modes and Binding Energies of Couples of Proteins and Nanoparticles

**Figure 1:**
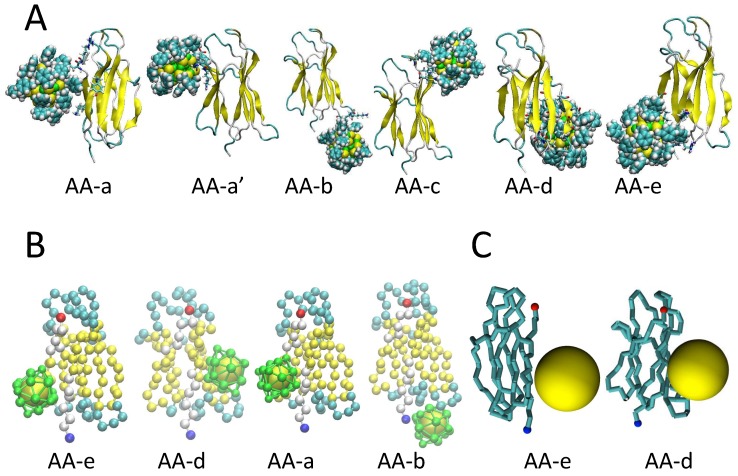
A pictorial description of binding modes. Most populated encounter complexes of β2μ (β2-microglobulin) on gold nanoparticle obtained by AA atomistic model (**A**) (reprinted with permission from Brancolini G., Toroz D., Corni S. (2014) *Nanoscale*, 6, 7903-7911) [7], with the CG (coarse-grained) model (**B**), and with the MS (mesoscale) model (**C**), respectively. The structures of representative complexes are ordered by decreasing cluster population. The protein backbone is shown in cartoon representation for the atomistic model and van der Waals representation for the CG and mesoscale models. In (**B**) and (**C**), each pose is labeled with the letter of the most similar AA atomistic pose.

The results of our CG and MS simulations (Figure 1B,C) were analyzed and compared with atomistic data (AA) on the same system (Figure 1A). In particular, by using BD rigid-body docking and classical MD, the molecular driving forces that guide the binding of β2μ to phenyl-functionalized gold NPs were studied. To establish the predictive power of our lower resolution protein minimalist model, we compared our results to AA simulations. Here, we performed rigid BD docking calculations, of the CG protein with respect to the CG NP model. We additionally compared with the MS model to investigate the role of the NP roughness and chemical functionalization, explicitly included in CG but not in MS. In Figure 1, a comparison between the binding modes obtained with the AA model (Figure 1A), the CG model (Figure 1B), and the MS (Figure 1C) is reported. 

The relative adsorption free energies of β2μ on the gold NP were computed for the structures resulting from the docking, see Table 1.

The binding energy of the protein-NP complexes was described by three main terms: van der Waals (vdW) energy described by site–site Lennard–Jones, E_LJ_, together with the non-polar desolvation energy of the complex, U_ds_ interactions, and the electrostatic interaction energy, U_EP_ (see Table 1).

The results showed that when the same BD docking protocol was applied to the CG model of the β2μ system with the gold NP, it yielded four different orientations (Figure 1B) with respect to the six orientations obtained with the AA model (Figure 1A). We wish to remark, that the CG model was able to capture the most representative complexes, namely complex AA-a and AA-b, from fully atomistic MD simulations, in which the binding is driven mainly by non-polar (hydrophobic) U_ds_ +E_LJ_ interactions and electrostatics is smaller. The CG model was able to capture also the complexes AA-e, AA-d found at the atomistic level. Four over six binding patches were obtained, revealing the capability of the CG model to capture the main AA complexes, as a result of the roughness of the NP surface, which was re-introduced including explicitly eighteen decorating spherical beads representing “surface ligands atoms” of 3.5 Å vdW radius, respectively. The first two most populated complexes, CG-a and CG-b in Table 1, had patches corresponding to the AA-e and AA-d atomistic complex, whereas complex CG-C and CG-D corresponded to AA-b and AA-a complex, respectively. A limitation of the CG model is due to the absence of the side chains, which affect the global orientation of the NP with respect to the protein. However, the main binding site for the anchoring of β2μ to the NP was captured. The strongest binding (CG-a, CG-c, and CG-d) was associated with the presence of positively charged residues contacting the surface of the negatively charged NP, with a small preference for LYS as already found in atomistic simulations. However, the predominant binding patches (see complexes CG-a and CG-b) were composed of a higher hydrophobic term, which reflected the high affinity of the protein for the phenyl-ligand hydrophobic patches on the surface of the NP.

**Figure 2:**
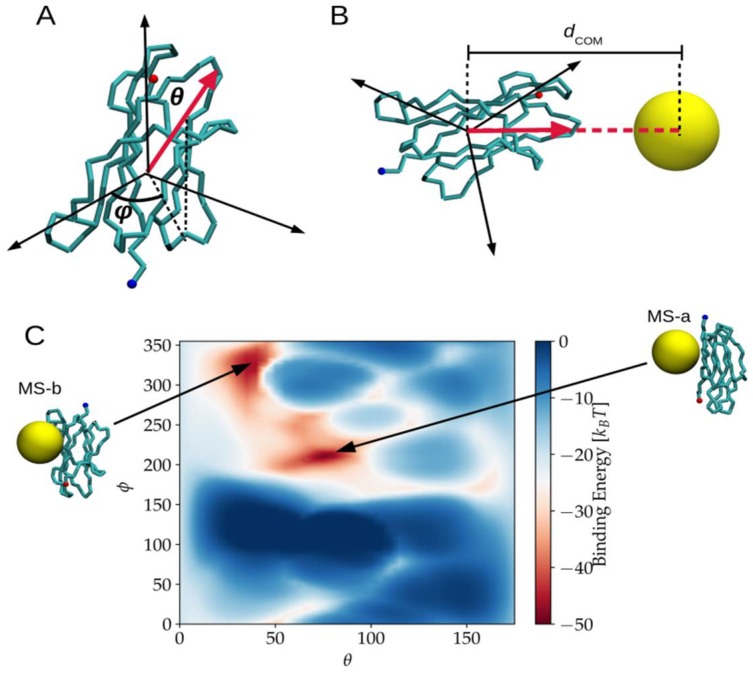
Schematic representation of the energy binding sampling and the binding energies results for the MS model. (**A**) A point on the surface of the protein can be represented by the pair of angles (*Φ,θ*). (**B**) The total potential energy is calculated for each pair if (*Φ,θ*) and a function of the distance from the center of the NP to the COM of the protein, *d_COM_*_,_ is known. The binding energy for each orientation is defined as the minimum of the total potential energy. (**C**) Binding energy map for β2μ interacting with Au_25_(S(CH_2_)_2_Ph)_18_^−^, Red areas show regions in the protein that bind more strongly to the NP.

For the MS model, two main binding patches were identified, as reported in Table 1, and showed by the two main minima in the binding energy map in Figure 2. This binding energy map also shows that big areas (meaning a big number of orientations) have binding energies lower than −30 k_B_T, which in practice means that more than just a few orientations give relatively strong adsorption. Hence, the MS model predicted that the protein would bind to NP at room temperature in various orientations. Regarding the two most favorable ones, these corresponded to the structures AA-d and AA-e obtained from AA simulation, but for the MS-a orientation, the weight of vdW and electrostatic potential contributions to the binding energies were not well captured. The binding energy of the MS-a orientation substantially overestimated the electrostatic. This might be due to the oversimplification of representing the whole distribution of charges of the NP as a single charge place at the center of the MS model. This finding highlights the importance of well representing the surface of the NP as ignoring its details can translate into proposing a model that does not represent the right chemical properties of the NP. Remarkably, by introducing the roughness of the NP surface in the CG model with respect to the simplest single sphere MS representation, we were also able to predict another representative binding patch, namely AA-b, which was not observed at the MS point of view. In this binding patch, the protein was contacting the NP through the N-Terminal tail and the BC-loop (including residue HIS31), which are known to play a crucial role in the protein-NP binding, as observed upon the refinement of the docking poses with MD simulations [7].

### 2.2. Simulation of Ensembles of Nanoparticles and Proteins

To shed light on the mechanisms of association of protein-NP in aqueous solution, BD simulations were carried out using 40 NP and 40 proteins that were initially randomly positioned (avoiding overlaps) in a rectangular box with periodic boundary conditions, with concentrations of 24 g/L and 46 g/L, respectively. A pH of 7 and an ionic strength of 30 mM was assumed [7].

**Figure 3:**
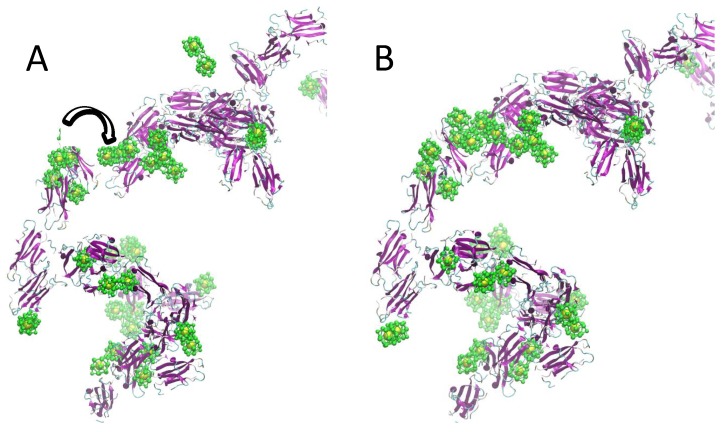
Formation of larger protein-NP aggregates during multiple solute simulations. (**A**) Formation of protein-NP oligomers with the NPs intercalating between protein-protein complexes especially by contacting the protein through the apical regions (**B**) The separated protein-NP complexes are associating into larger aggregates through the NP-NP interaction occurring via protein apical region already covered with NPs. Protein all-atom reconstruction was performed with PULCHRA [25,26].

Looking at the encounter complexes identified in the simulations, we could hypothesize the following mechanism for the formation of larger protein-NP complexes: the initial formation of smaller protein-NP complexes (see Figure 3A) is followed by the association into larger aggregates occurring through the NP-NP association (see Figure 3B). 

**Figure 4:**
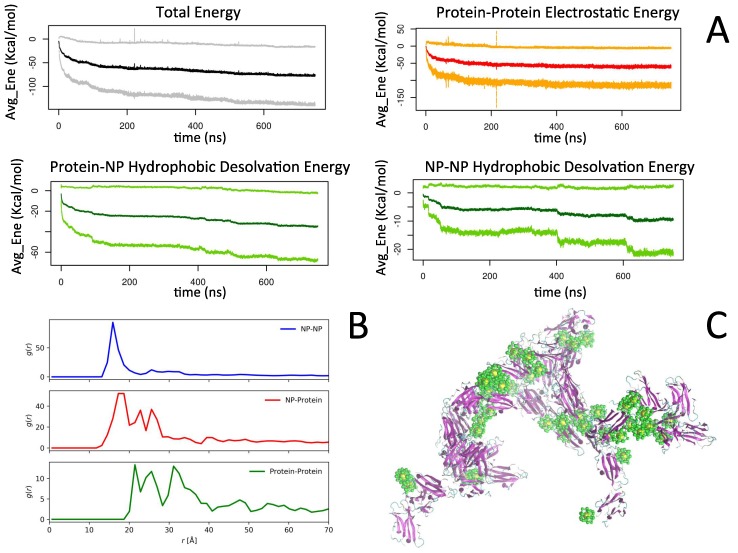
Summary of the CG simulations of ensembles of nanoparticles and proteins. (**A**) Energies terms contributing the most to the Total Binding Energy (in kcal/mol) collected during the dynamics of 1 μs of simulation at 300 K. (**B**) Radial Distribution Function of the pairs NP-NP, NP-Protein, and Protein-Protein at the CG level. (**C**) Snapshot of the final configuration. Protein all-atom reconstruction was performed with PULCHRA [25].

The formation of larger complexes is stabilized mainly by non-polar hydrophobic protein-NP interactions and protein-protein electrostatic interactions, but at longer timescale is mediated by the NP-NP interactions (see Figure 4A).

As a result of the interplay between different competing forces, we observed an initial aggregation of protein with NP, which once formed, remained stable during the entire length of the simulations. The NP-protein binding was able to interfere with the protein-protein binding, blocking protein sites for the binding with another protein, thus leading to a potential protein-protein aggregation inhibition depending on the interaction strength between the NP and the protein atoms. Given to the hydrophobic character of the NP, an additional competing force, namely NP-NP hydrophobic interaction, provided a picture in which the initial NP-protein complexes were associating into larger aggregates via NP-NP hydrophobic interactions (Figure 4C). Our study underlies the crucial role of the relative concentrations between proteins and NPs, on amyloid aggregation. Therefore, to theoretically characterize the effects of NPs on amyloid aggregation, more investigations need to be conducted, and it is necessary to study a wide range of NP-protein ratios. This is the subject of an upcoming paper.

**Figure 5:**
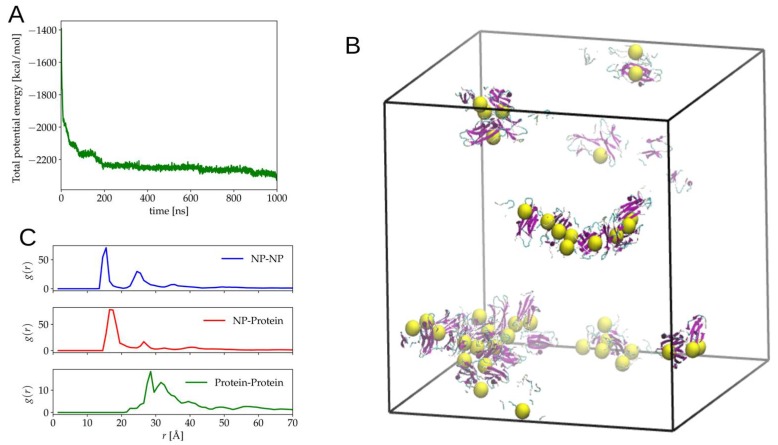
(**A**) Time evolution of total potential energy dynamic simulations using the MS model. (**B**) Snapshot of the final configuration. (**C**) The radial distribution function of the pairs NP-NP, NP-Protein, and Protein-Protein. Protein all-atom reconstruction was performed with PULCHRA [26].

Using the MS model, we again simulated the dynamics of 40 NP and 40 proteins in the same conditions as the simulations performed with the CG model. The total potential energy of the system as a function of time is shown in Figure 5A. 

For the first 50 ns, we observed a quick drop of the potential energy indicating a fast formation of aggregates. After this rather fast process, the time evolution of the potential energy showed step like drops, which were due to the combination of clusters. The final state of the system is presented in Figure 5B, and it shows that the aggregates were mainly composed of a cluster of NP and proteins attached to these clusters. To further characterize the final state, we calculated the pair radial distribution functions, *g(r)*, of the NP-NP, NP-protein, and protein-protein for the CG model (Figure 4B) and the MS model (Figure 5C). In the case of the protein, we used the COM of the protein for the calculation of *g(r)*. The NP-NP *g(r)* showed the first peak at ~15 Å for both models which corresponded roughly to the diameter of the NP, which indicated the formation of a cluster of two NPs. By eye inspection of the trajectories, it was confirmed that cluster of two or more NPs was present for both models, but their structures were qualitatively different. In the CG model, the aggregates tended to be compact and spherical, while for the MS model, they appeared more linear. This observation could be confirmed by the *g(r)*’s. For the CG model, a rather shallow peak was observed at two times the diameter. In contrast, the MS model presented a sharp second and the third peak at two and three times the diameter of the NP, which indicated the formation of NP-NP linear clusters of three and even four NPs. In the case of NP-protein interaction, from the binding energy calculations for the MS model, we observed that the most favorable binding orientation was of the type of what can be called side-on (see Figure 2C), i.e., if one represents the protein as a flat rod, the most favorable configurations in which the protein attaches to the NP are on the flat faces. The NP-protein *g(r)* showed the first peak at ~17 Å, which corresponded to these orientations as the width of the protein, plus the radius of the NP was approximately this value. A small secondary peak was also observed and might reflect the presence of a small number of other configurations. For the CG model, the peak at ~17 Å was also observed, but other two relevant peaks for larger *r* were also present, indicating that for the CG model, other modes of attaching apart for the side-on configuration were also important. For the CG model, the protein-protein *g(r)* presented a series of peaks starting at ~21 Å, indicating the presence of protein-protein complexes. On the other hand, the protein-protein *g(r)* for the MS model presented the first peak at ~28 Å, which suggested that protein-protein binding was due to a different configuration than the ones obtained with the CG model and probably mediated by NPs. It is important to notice that the comparison of the dynamic results was done based on a single trajectory for each method. Hence, further statistical sampling is needed to better compare the formation of the aggregation process and the structure of the aggregates.

## 3. Materials and Methods

**Figure 6:**
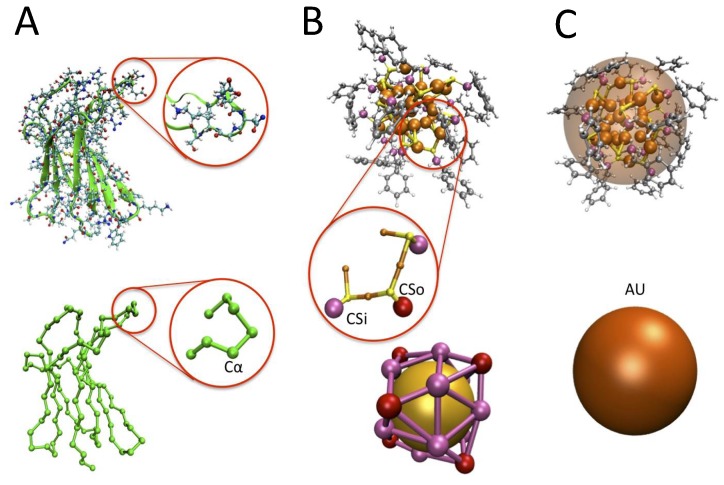
Coarse-graining procedures. (**A**) atomistic and CG-minimalist model of the β2μ (the zoomed-in detail reports a loop, with the atomistic detail and the Cα beads for the CG case in green) (**B**) atomistic and CG model for the NP. The detail reports the Au (orange) coordinated S atoms (yellow) with the first C atom of the functionalizing chain, colored according to the two possible different coordinations of their bound S, and named CSo (outer) and CSi (inner), respectively. (**C**) the mesoscale model for the NP, i.e., a single larger bead.

In this work, several different representations of the molecular species involved were used (see Figure 6): the β2μ was represented using a Cα-based minimalist CG model; the NPs were represented at two levels of coarse-graining, i.e., with a sphere for the gold core and single smaller spheres for the 18 functionalizing groups (in yellow and green in Figure 6), or with a single larger sphere representing the whole NP. Atomistic MD simulations were also performed for both systems, separated and in interaction. Details of the models and setup are reported in the following subsections. We remark that the optimization and parameterization of the two models is already, at least partially, an original contribution of this work.

### 3.1. The Protein Minimalist Models

The minimalist representation of the protein has been previously used and validated for several proteins in different situations [15,16]. The protein is an un-branched chain of beads, representing as single amino-acids and placed on the Cαs (Figure 6A). In general, the force field (FF) consists of bonded and non-bonded terms similar to those present in standard atomistic FFs, though using less simple functional forms, parameterized based on a combination of structure-based and statistics-based parameterization [27]. The latest version includes a set of numerical pair potentials (including both the hydrophobic/steric component and electrostatics, and with implicit solvent) for the non-bonded part, capable of reproducing the different amino acids aggregation tendencies [17], even in the presence of other elements, such as cytoplasm “crowders” molecules [28]. In this work, while we wanted to maintain the amino-acid specificity of the interaction, we also needed to separate electrostatics to treat different ionic strength of the solution. Therefore, we adopted a standard decomposition of the hydrophobic/steric (*hs*) and electrostatic interactions (*el*)

(1) $$U=\sum_{i>j}u_{\varepsilon ,r_{ij}^{0}}^{hs}(r_{ij})+\sum_{i>j}u_{q_{i}q_{j},\lambda }^{el}(r_{ij})$$

and used analytical forms for the two components. Additionally, to use the standard implementation of rigid Brownian dynamics and Rigid Docking software packages, in this work, we neglected the internal dynamics of the protein (and NPs).

### 3.2. The Nanoparticle Models and Its Interaction with the Protein

The NP CG model (Figure 6B) was built to be compatible with the protein minimalist model. Taking as a reference the Au_25_(S(CH_2_)_2_Ph)_18_^−^, we used a single bead (named Au) for the gold core and 18 beads representing the functionalizing chemical groups, named CS. By placing the CS bead on the C atom bound to the bridging S, we obtained, for the (CH_2_)_2_Ph group, a representation analogous to the protein phenylalanine, which allowed to use similar parameters for the CS and Cα phenylalanine beads. At variance with those, however, the CS beads bore a negative charge, which needed to overbalance the positive Au charge, leading to a total charge of −1. Also, the 18 CS beads belonged to two different groups bound to S with different coordination, namely 12 inner beads (CSi) and 6 outer beads (CSo). These might bear different charges. The charges optimization procedure is reported in the next sections, as the optimization of their relative location.

Besides the CG representation, in this work, we also used a coarser (mesoscale MS) representation, with the NP represented by a single bead, bearing the whole mass and charge of the NP. It was expected that this representation could account only roughly for the binding mode geometry of the protein to NP; however, it was much less computationally expensive and required the optimization of fewer parameters. Furthermore, as the simulated NP is highly hydrophobic, the interaction parameters for the NP-P can be chosen by analogy with [24,29]. In this model, the NP and the amino acids were characterized by a hydrophobicity index, which can be defined in different ways, and only one single free parameter was needed to parameterize the FF. Following the same scheme as in [24,29], electrostatics was represented with a Debye-Hückel potential, to account for the ionic strength of the solution. The functional forms and other details are reported in the Supplementary Materials (Section S2). The comparison between the results of the CG minimalist protein model with the CG and MS models for the NP is the focus of this work.

### 3.3. Optimization of the Parameters

The parameters to be optimized were the vdW radii, the hydrophobicity scales, and the charges. For the NP CG model, additionally, also the relative bead location need to be assigned. As far as possible, the parameterization was based on a combination of physics-based previously optimized parameters (e.g., the hydrophobic interaction). Missing parameters were obtained from atomistic simulations.

**Figure 7:**
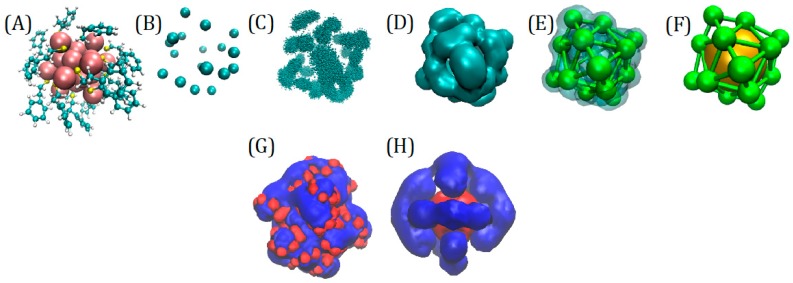
(**A**) Atomistic structure of the NP, (**B**) CG representation (only CS beads), (**C**) space clustering of the CS beads, (**D**) volume map based on CS positions, (**E**) CS location based on the volume map, (**F**) Complete CG model with AU. Representation of the electrostatic potentials around the NP: (**G**) atomistic model based on RESP (Restrained Electrostatic Potential) from GolP force field atom-based charges and (**H**) 19 beads CG model with charges obtained as sum of the constituent RESP atom charges (UHBD, University of Houston Brownian Dynamics, isosurfaces drawn at positive +0.5 kcal/e (red) and negative −0.5 kcal/e (blue) values).

For the NP-CG model, one needed to, first of all, fix the relative position of the AU bead and the 18 beads corresponding to the functional groups. As mentioned before, their representative atoms were chosen to be the C bound to S (CS). The procedure to find their optimal location is described in Figure 7. Using a 500 ns atomistic simulation of a single fully hydrated nanoparticle, we first aligned the NP atomistic structure to the gold core atoms (Figure 7A) and then “coarse-grained” the trajectory leaving only AU and CS (Figure 7B). Subsequently, we built a volume map from the space superposition of CSs and found the centroids of the 18 space clusters [30] (Figure 7C–E), which are as CS beads locations. Further details are reported in the Supplementary Materials (Section S3).

Remarkably, the location of the beads reflects the symmetry of the gold core, the outer 6 CSo located at the vertices of an octahedron, and the 12 CSi approximately at the center of the edges. This naturally suggests that the model should have three different charges, for Au, for CSo, and CSi. The procedure for the charge assignment is also illustrated in Figure 7. The charges on the CG sites had been taken as the sum of the constituent atomic charges of the atoms belonging to the beads, namely the RESP atomic charges [31,32] based on ab initio calculations (details reported in the Supplementary Materials. The derivation of the CG charges based on the atomistic components [31,32,33] was able to predict the dependence of CG charges on the bead type (gold or ligand) and NP symmetry (Figure 7H). The comparison of atomistic with CG electrostatic potential showed that the general shape of the iso-surfaces was preserved (Figure 7H), while the atomistic detail was lost. The CG model was able to reproduce the global net prevalence of negative character (in blue), and some positive areas (in red), and reproduce the relevant features of the electrostatic field. The assignment of the bead masses might be done simply assigning to each bead the sum of the masses of its component. However, this can be shown to bring an unbalanced inertia momentum because the very massive gold atoms are concentrated in a single point-like bead in the model. We corrected this by making CS beads heavier (and AU lighter), as outlined in the Supplementary Materials (Section S1).

For the NP-protein short-range interactions, two classes of parameters were needed, namely the equilibrium distances (or vdW radii or diameters or σ) and the hydrophobic interaction strength ε. These are based on atomistic simulations. While the ε value was initially taken from that of phenylalanine, the σ values of the interaction were tuned until the radial distribution functions (RDF) of CS-CS, CS-AU, and AU-AU from 500 ns of standard MD of 2 NPs atomistic (see SI) and CG simulations were matched. The σ values for the protein beads interactions were taken from previous works [7] For the CS-protein interactions, the linear combination rule was first considered and shown to give a good match with the RDF evaluated from atomistic simulations of single NP-protein interaction. In the case of the mesoscale model, the parameter ε for the phenyl rings of the NP was first assigned as that of phenylalanine according to the hydrophobicity scale previously defined [24] The optimized parameters are reported in Table S2 in the Supplementary Materials, together with other details about the parameters optimization.

### 3.4. Simulations Methodology and Setup

#### 3.4.1. Setup of the Atomistic Simulations

A set of reference atomistic data of the isolated NP and of a dimer of two interacting NPs was used for the parameterization CG model for the NP. Atomistic simulations were performed with the Gromacs 4.6.7 [34] package implementing the GolP force field [35], which includes specific parameters for the thiol-protected AuNP [7]. The system was solvated with the SPC/E water model. Classical MD simulations were performed at constant volume and room temperature, using periodic boundary conditions (Particle-Mesh-Ewald algorithm within simulation box size 80 Å × 80 Å × 80 Å). A 2 fs integration time step was used, constraining the hydrogen bond lengths with the LINCS algorithm. For the optimization of protein-NP CG model, we used a series of independent MD simulations of β2μ/NP interacting with different relative orientations previously performed with the same protocol [4].

#### 3.4.2. Setup for CG Rigid Docking Simulations

SDA7 [36] for the rigid docking of molecules code was adapted from atomistic to CG representation. Very briefly, SDA7 evaluates the free energy of the system by summing four types of interaction described as “charges” of beads on solute 1 interacting with a potential grid of solute [37]: (i) the Coulombic electrostatic interaction, evaluated as the bead effective charge of solute 1 interacting with the potential grid of solute 2 (averaged with exchanged terms), (ii) the electrostatic desolvation interaction, evaluated similarly to term (i) but using the desolvation grid and depending on the squared effective charges, (iii) the non-polar part of desolvation energy, evaluated using the solvent-accessible surface of solutes interacting and the non-polar desolvation grid, (iv) the repulsive softcore term, evaluated using the Lennard-Jones grid. The SDA interaction potentials are then

(2) $$\begin{array}{cc} \Delta G & =\frac{1}{2}\sum_{i2}\phi_{el1}q_{i2}+\frac{1}{2}\sum_{j1}\phi_{el2}q_{j1}+\sum_{i2}\phi_{edesolv1}q_{i2}{}^{2}+\sum_{j1}\phi_{edesolv2}q_{j1}{}^{2}+\sum_{m2}\phi_{npedesolv1}SASA_{m2}+\\ & +\sum_{m1}\phi_{npedesolv2}SASA_{m1}+\frac{1}{2}\sum_{i2}E_{lj1}+\frac{1}{2}\sum_{j1}E_{lj2} \end{array}$$

where $\phi_{el1}$ is the electrostatic potential of solute 1 and $q_{i2}$ is the effective charge of bead *i* on solute 2 ($\phi_{el2}$ is the electrostatic potential of solute 2 and $q_{j1}$ is the effective charge of bead *j* on solute 1), $\phi_{edesolv1}$ is the electrostatic desolvation potential of solute 1 ($\phi_{edesolv2}$ is the electrostatic desolvation potential of solute 2), $\phi_{npedesolv1}$ is the non-polar burial potential of solute 1 and $SASA_{m2}$ is the solvent-accessible surface area of bead *m* on solute 2 ($\phi_{npedesolv2}$ is the non-polar burial potential of solute 2 and $SASA_{m1}$ is the solvent-accessible surface area of bead *m* on solute 1), $E_{lj1}$($E_{lj2}$) is a softcore repulsive potential of solute 1 (solute 2). Effective charges, vdW radii, and specific surface desolvation and hydrophobic energy were involved. The adaption of the AA method to the CG models consisted of the re-optimization of the VdW radii and charges. Details are reported in the SI.

In addition to two solute docking, multiple solute BD simulations were performed with SDAMM [38] of a mixture of 40 NPs and 40 proteins. Simulations at 300 K, 1 μs long were performed on each system, using 0.4 ps as timestep. Thermalization was checked through the control of the convergence of average energies and radial distribution functions, and the run length was shown to be sufficient for equilibration, according to these criteria. The dumping frequency of position and orientation of proteins and energies was 0.5 ns. 

The self-diffusion translational and rotational coefficients were calculated for the protein and NP with HYDROPRO [39] and included in the calculation.

#### 3.4.3. Dynamics MS Simulations Setup

Dynamic simulations using the MS model were performed using HOOMD [40,41]. The box size and number of NPs and proteins were the same as in the CG simulations. A Langevin thermostat with a friction coefficient of 0.001 ps^−1^ was used, and the time step was set to 0.15 ps. NPs were assigned a mass of 7.4 kDa, while all amino-acids were assigned a mass of 0.12 kDa. This value comes from dividing the total mass of the protein (12 kDa) by the number of residues of the protein (99). The proteins were kept rigid during the simulation using the rigid body tool implemented in HOOMD [42].

#### 3.4.4. NP-Protein Binding Modes and Energies Evaluation in the MS System

Because in the MS model, the NP does not have any surface structure, the characterization of the binding modes and energy can be characterized using only two coordinates, namely the azimuthal and polar angles (*θ,φ*) described by the vector connecting the centre of mass (CM) of the protein and NP with respect to a fixed orientation of the protein itself [24] (See Figure 4A). For any pair (*θ,φ*), the point in the surface was faced to the surface of the NP, and the total potential energy was calculated as a function of the distance between the COM of the protein and the center of the NP denoted as *d_COM_* (see Figure 4B). The binding energy for a particular orientation (*θ,φ*) was calculated as the minimum of total potential energy, U_min_(*θ,φ*). Once a set of N orientations were sampled, the average binding energy E_b_ was calculated as the Boltzmann average,

(3) $$E_{b}=\frac{\sum_{i,j}^{N}P_{ij}U_{\min}(\phi_{i},\theta_{j})}{\sum_{i,j}^{N}P_{ij}}$$

where *P_ij_* is the Boltzmann weighting factor. In practice, we systematically sampled *φ* from 0 to 350° in steps of 10° and *θ* from 0 to 170° in steps of 10°.

## 4. Conclusions

We have developed a low-resolution CG model to describe the β2-microglobulin interaction with coated gold nanoparticles and compared results obtained with two different representations of the NPs, i.e., the CG level with explicit representation of the chemical coating and the MS model, representing the NP as an unstructured sphere. Our results showed that neglecting the surface roughness and the chemical details of specific NPs, important properties contributing to the protein association to the NP could be omitted. For example, our dynamic simulations showed that the structure of aggregates differed for the MS and the CG models. In particular, the MS NPs tended to form linear aggregates, while the CG NPs formed more globular ones. In part, this was because the simplified spherical model of the NP was not able to capture the variability of the different possible orientation of the NP-protein and NP-NP binary. At the same time, we showed that the combination of a CG model for the NP and minimalist model for proteins included all the crucial elements for a low-cost and large scale molecular dynamics simulations of the interaction of proteins with NPs, that could be used for systematic studies of NP-protein solution dynamics. In our opinion, the minimalist model for proteins and the CG model for the NPs are the minimal resolution representations including all the crucial elements, such as the specificity of chemical decoration and the coherency between the NPs and proteins representation. Additionally, we proposed a parameterization strategy involving atomistic simulations, which is easily extensible to different chemical functionalization (possibly introducing different global charges in the NPs) and different NPs size, being the choice of decorating bead location (CS) independent of the decorating moiety.

We envision that our approach could be used to unravel the different biophysical contributions to protein motion and interaction in NP environments by systematically varying NP properties, such as molecular weight, size, shape, and electrostatic interaction. This work opens the way to a fast and systematic study of the effect of size and decoration of NPs over therapeutic efficiency.

## Figures and Tables

**Table 1:** Summary table of the results for protein-NP (nanoparticle) binding modes/energies.

Label	RelPop ^(a)^	U_rep_^(b)^	E_LJ_ + U_ds_^(c)^	U_ep_^(d)^	Spread ^(e)^	Contact Residues ^(f)^
CG-a^(AA-e)^	59	−44.1	−30.3	−13.8	10.4	THR4, PRO5, LYS6, LEU87, LYS91, VAL93
CG-b^(AA-d)^	23	−43.6	−35.0	−8.5	9.3	LYS6, ILE7, GLN8, TYR26, VAL27, SER28, SER55, SER57, TYR63, LEU64, LEU65
CG-c^(AA-b)^	12	−45.0	−34.6	−10.5	0.6	NTR1, ARG3, HIS31, PRO32, TRP60, SER61
CG-d^(AA-a)^	6	−46.2	−30.9	−15.3	15.4	THR4, PRO5, LYS6, VAL82, HIS84, ASN83, THR86, LEU87, GLN89, LYS91, VAL93
MS-a^(AA-e)^	(*)	−48.8	−28.7	−20.1	(**)	VAL93 LYS91 LEU87 PRO5 THR4 LYS6 ILE7
MS-b^(AA-d)^	(*)	−45.9	−40.9	−5.0	(**)	LEU64 TYR63 SER57 SER55 SER28 VAL27 TYR26 GLN8 LEU65 SER52
AA-a	28	−48.0	−27.4	−20.6	2.2	TYR10, LYS91, ASP96, ARG97
AA-a’	28	−44.5	−27.5	−17.1	15.2	GLY43, GLU44, ARG45
AA-b	18	−41.9	−31.7	−10.2	2.67	LYS58, ASP59, TRP60
AA-c	16	−42.4	−48.3	5.9	7.9	MET99, HIS13, PRO14, GLU16, LYS19
AA-d	4	−47.3	−44.5	−2.8	1.8	SER33, ASP34, ILE35, LEU54, ASP53, LEU64, GLU36, VAL37, HIS51, TYR66
AA-e	6	−46.5	−49.4	2.8	1.4	THR86, LEU87, SER88, GLN89, LYS91

^(a)^ Relative population of this cluster ^(b)^ U_rep_: total interaction energy of the representative of the given cluster in kT with T= 300 K, ^(c)^ E_LJ_: Lennard-Jones energy term for the representative complex, U_ds_: non-polar (hydrophobic) desolvation energy of the representative complex, in kT, ^(d)^ U_ep_: total electrostatic energy of the representative complex, in kT, ^(e)^ Spread: (Root Mean Square Deviation) of the structures within the cluster with respect to the representative complex, Å, ^(f)^ Contact Residues: Residues at 5.5 Å distance (CG and MS) and at 3.5 Å distance (AA) from the NPs in the final most representative patch, (*) See binding energy maps in Figure 2 for RelPop (**) not available for mesoscale calculation.

## Supplementary Materials

Supplementary materials can be found at https://www.mdpi.com/1422-0067/20/16/3866/s1.
