# Supplementary material for: Low-Resolution Models for the Interaction Dynamics of Coated Gold Nanoparticles with β2-microglobulin
Source: Int J Mol Sci. 2019 Aug 8;20(16):3866. doi: 10.3390/ijms20163866 (PMC6719018; doi:10.3390/ijms20163866)
Supplement: Supplementary file 1 [file ijms-20-03866-s001.pdf]

International Journal of
Molecular Sciences

MDPI

Article

Supporting Information of "Low-Resolution Models for the Interaction Dynamics of Coated Gold Nanoparticles with β2-microglobulin"

S.1 Building the CG model of the Nanoparticle

In this section, some details about the parameterization of the NP-CG model are reported.

Evaluation of the effective masses of NP beads

The total mass of the NP was M=7394 au. The total rotational inertia roughly evaluated from that of a solid sphere of radius R (roughly corresponding to that of the NP, see below for its value) was $I=(2/5)MR^2$. The following equations were then to be solved

$$M^{\mathrm{CG}} = 18m_{\mathrm{CS}} + m_{\mathrm{AU}} = M \quad I^{\mathrm{CG}} = 18m_{\mathrm{CS}}R_{\mathrm{CS}}^2 = \frac{2}{5}\mathrm{MR}^2 \quad \rightarrow 18m_{\mathrm{CS}}R_{\mathrm{CS}}^2 = \frac{2}{5}(18m_{\mathrm{CS}} + m_{\mathrm{AU}})R^2$$
$$R = \alpha R_{\mathrm{CS}} \quad \rightarrow \quad m_{\mathrm{CS}} = M\frac{\alpha^2}{45} \qquad m_{\mathrm{AU}} = M\left(1 - \frac{2}{5}\alpha^2\right) \tag{1}$$

being m_{CS} the mass of the CS beads and m_{AU} that of the Au bead, and R_{CS} the location of CS beads with respect to the central Au (AU bead do not contribute to rotational inertia because it is in the center). The ratio α between the NP radius and the location of R_{CS} was ~1.35, according to the radial distribution of the NP atoms, which resulted in the masses values reported in Table S.1. With respect to the masses obtained summing the values of component atoms, the m_{CS} was little more than double, and m_{AU} was little less than half.

Table S1. Table with CG (coarse-grained) model for the NP (nanoparticle) and mass parameters.

	**Name**	**Composition**	**Location**	**Mass**	
				**Real**	**Effective**
**Gold core**	AU	24 Au atoms	Cluster center	4922	1994
**Functional group inner**	CSi	Sulfur and functional group, internal	Carbon bound to Sulfur	137	300
**Functional group outer**	CSo	Sulfur and functional group, external	Carbon bound to Sulfur	137	300

S.2. Description and parametrization of Mesoscale (MS)

For the MS model, as explained in the main text, the NP representation consisted of a single bead while, as in the CG model, the protein was represented by a single bead per residue. The van der Waals (vdW) interactions $U^{hs}{}_{ij}$ between two beads *i* and *j* were adapted from the modified 12-6 Lennard-Jones potential reported in [1] and are given by:

$$U_{ij}^{hs}(r_{ij}) = \begin{cases} 4\epsilon_{ij}\left[\left(\frac{\sigma_{ij}}{r_{ij}}\right)^{12} - \left(\frac{\sigma_{ij}}{r_{ij}}\right)^{6}\right] + \epsilon_{ij}(1-\delta_{ij}), & r_{ij} < r_{c,ij}, \\ 4\epsilon_{ij}\delta_{ij}\left[\left(\frac{\sigma_{ij}}{r_{ij}}\right)^{12} - \left(\frac{\sigma_{ij}}{r_{ij}}\right)^{6}\right], & r_{c,ij} \le r_{ij} \le r_{\text{cut}}, \\ 0, & r_{ij} > r_{\text{cut}}, \end{cases} \quad (2)$$

where r_{ij} is the center-to-center distance between the beads *i* and *j*, ε_{ij} is a scaling factor that sets the strength of the interaction, σ_{ij} is the average van der Waals diameter of bead *i* and *j*, $\sigma_{ij} = 0.5(\sigma_i + \sigma_j)$, δ_{ij} is the combined hydrophobicity index of bead *i* and *j* and, following the Lorentz-Berthelot mixing rules, is given by $\delta_{ij}=(\delta_i\,\delta_j)^{1/2}$, and $r_{c,ij}$ is the position of the minimum of the pair potential, $r_{c,ij} = 2^{(1/6)}\,\sigma_{ij}$. The hydrophobicity nature of each bead is characterized by its hydrophobicity index, which is in the range 0 to one, where the most hydrophilic bead is assigned a value of 0 while one is assigned to the most hydrophobic one. For the amino acids, we used the hydrophobicity index reported by Bereau and Deserno [2], which was calculated based on Residue-Residue contact potentials [3]. This way, in our model, 0 was assigned to the most hydrophilic residue (LYS), and an index one to the most hydrophobic one (LEU). For the NP, as it can be considered as a highly hydrophobic bead, we assigned it a hydrophobicity index of one.

For the electrostatic interaction, we employed a Debye-Hückel potential given by:

$$U_{ij}^{el}(r_{ij}) = C_{ij}\lambda_B k_B T q_i q_j \frac{\exp(-r_{ij}/\lambda_D)}{r_{ij}} \quad (3)$$

where C_{ij} is a scaling parameter for the electrostatic interaction between beads *i* and *j*, $\lambda_B = e^2/(4\pi\varepsilon_0\varepsilon_r k_B T)$ is the Bjerrum length, k_B is the Boltzmann constant, T the temperature, ε_0 the dielectric permittivity of vacuum, ε_r the relative dielectric permittivity of water, q_i the charge of bead *i*, q_j the charge of the bead *j*, and λ_D is the Debye length (defined through $\lambda_D^{-2} = 8\pi\eta\lambda_B c_0$, with c_0 is the background electrolyte concentration).

For the interactions defined in Eqs. 2 and 3, the free parameters ε_{ij} and C_{ij} have to be determined. Notice that not all possible combinations of beads types have to be defined, i.e., the NP and the 20 amino-acids (AA), but it is only necessary to determine a single value for the NP-NP, NP-AA, and AA-AA interactions. For the NP-NP interaction, the value of ε_{ij} was obtained by matching the minimum of the potential of mean force (PMF) calculated for the interaction of two NPs computed from atomistic simulations. From the same PMF, we also determined the value of σ for the NP. The values obtained were: $\sigma_{NP,NP}$ = 12.7 Å and $\varepsilon_{NP,NP}$ = 49.0 k_BT. For the AA-AA interaction, the strength of the vdW interaction was parameterized to match the LEU-LEU interaction reported by Kim and Hummer [4], which is modeled by a 12-6 Lennard-Jones potential. In**Error! Bookmark not defined.**, six values of the interaction strength are reported to match experimental data for six different proteins. In this work, we used the average of these six values, obtaining $\varepsilon_{AA,AA}$ = 0.2 k_BT. For the NP-AA, the value of $\varepsilon_{NP,AA}$ was selected to match the Boltzmann average binding energy of the most favorable complexes obtained by the atomistic simulations reported by Brancolini et al. [5]. In practice, the value of $\varepsilon_{NP,AA}$ was changed systematically, and the average binding energy was calculated, as explained in the main text. With a $\varepsilon_{NP,AA}$ = 3.0 k_BT, we obtained average binding energy of –47.7 k_BT, which is close to the Boltzmann average (–47.5 k_BT) of the binding energies reported in [5]

For simplicity, we chose that all AA have the same size, and from radial distribution functions from atomistic simulations of the binding of NP-proteins, we estimated $\sigma_{AA,NP}$ =10.7 Å . From $\sigma_{AA,NP}$ = ($\sigma_{AA,AA}$ + $\sigma_{NP,NP}$)/2, we obtained that $\sigma_{AA,AA}$ =8.7 Å.

For the electrostatic interaction, Eq. 3, the parameters defined by the medium, water with monovalent electrolyte concentration of 30 mM were: λ_D =18.0 Å and λ_B =7.0 Å. Residue charges at these conditions were +*e* for LYS and ARG, −*e* for ASP and GLU, and +0.5*e* for HIS. The rest of the residues were neutral. For the NP bead, the charge was set to the total charge of the NP, i.e., –1*e*. Now, for the AA-AA, $C_{AA,AA}$ was set to one as this interaction had the right energy scaling. For the NP-NP and AA-NP, the electrostatic interaction had to be scaled as placing a point charge at the center of the NP resulted in an underestimation of the interaction as the screening length was of the same order as the size of the NP. To estimate the correction, we performed a calculation of the electrostatic potential using the CG model and a test bead representing an AA. The test bead was placed close to the surface of the CG NP, and then it was moved radial outward to calculate the electrostatic potential as a function of the distance between the test bead and the center of the NP. We averaged over 50 different initial configurations and found that a correction factor of 25 was needed to match the CG potential to the MS one. In this way, we defined $C_{AA,NP}$ = $C_{NP,NP}$ =25.

S3. Summary table of the interaction parameters

Table S2. Table with CG model optimized parameters.

Bead Type	vdW radii	Charges on Beads	CG-Hydrophobicity [1]
CAu	4.00	2.525	Y=0.87
oCS	3.50	–0.128	Y=0.87
iCS	3.50	–0.230	Y=0.87
CA	4.50	+1/0/–1	Y=0.5

(1) For the construction of the non-polar desolvation grid, a set of input parameters in SDA7 specified the region where the potential was defined and the scaling factor for the calculated potential. The procedure assigned a value of gamma to all points within distance a (in Å) from the surface of the solute, zero if a point was further than b (in Å) from the surface, and a linearly interpolated value if a point was in between a and b. In SDA, the solvent accessibility values of the atoms of one solute were multiplied by the nonpolar desolvation values of the other solute. In the CG model, the value of gamma was set to 0.5 for the protein, and 0.87 for the NP.

References

1. Lopez, H.; Lobaskin, V. Coarse-grained model of adsorption of blood plasma proteins onto nanoparticles. *J. Chem. Phys.* 2015, *143*, 243138
2. Bereau, T.; Deserno, M. Generic coarse-grained model for protein folding and aggregation *J. Chem. Phys*. 2009, *130*, 235106
3. Miyazawa, S.; Jernigan, R. L. Residue – Residue Potentials with a Favorable Contact Pair Term and an Unfavorable High Packing Density Term, for Simulation and Threading. *J. Mol.Biol.* 1996, *256*, 623-644
4. Kim, Y.C.; Hummer, G. Coarse-grained models for simulations of multiprotein complexes: application to ubiquitin binding. J. Mol. Biol. 2007, 375, 1416-1433.
5. Brancolini, G.; Toroz, D.; Corni, S. Can small hydrophobic gold nanoparticles inhibit β2-microglobulin fibrillation? *Nanoscale* 2014, *6*, 7903-7911.
